# Near-Infrared Spectroscopy Coupled Chemometric Algorithms for Rapid Origin Identification and Lipid Content Detection of *Pinus Koraiensis* Seeds

**DOI:** 10.3390/s20174905

**Published:** 2020-08-30

**Authors:** Hongbo Li, Dapeng Jiang, Jun Cao, Dongyan Zhang

**Affiliations:** College of Mechanical and Electrical Engineering, Northeast Forestry University, Harbin 150040, China; lhb@nefu.edu.cn (H.L.); jiangdapeng187@nefu.edu.cn (D.J.); nefuzdhzdy@nefu.edu.cn (D.Z.)

**Keywords:** NIR spectroscopy, *Pinus koraiensis* seeds, chemometric algorithms, preprocessing, feature selection

## Abstract

Lipid content is an important indicator of the edible and breeding value of *Pinus koraiensis* seeds. Difference in origin will affect the lipid content of the inner kernel, and neither can be judged by appearance or morphology. Traditional chemical methods are small-scale, time-consuming, labor-intensive, costly, and laboratory-dependent. In this study, near-infrared (NIR) spectroscopy combined with chemometrics was used to identify the origin and lipid content of *P. koraiensis* seeds. Principal component analysis (PCA), wavelet transformation (WT), Monte Carlo (MC), and uninformative variable elimination (UVE) methods were used to process spectral data and the prediction models were established with partial least-squares (PLS). Models were evaluated by $R^{2}$ for calibration and prediction sets, root mean standard error of cross-validation (RMSECV), and root mean square error of prediction (RMSEP). Two dimensions of input data produced a faster and more accurate PLS model. The accuracy of the calibration and prediction sets was 98.75% and 97.50%, respectively. When the Donoho Thresholding wavelet filter ‘bior4.4’ was selected, the WT–MC–UVE–PLS regression model had the best predictions. The $R^{2}$ for the calibration and prediction sets was 0.9485 and 0.9369, and the RMSECV and RMSEP were 0.0098 and 0.0390, respectively. NIR technology combined with chemometric algorithms can be used to characterize *P. koraiensis* seeds.

## 1. Introduction

*Pinus koraiensis* is a rare and valuable species and a National Class II protected wild plant in China [1]. It is only found in the area between the Changbai Mountains and the Xiaoxingan Mountains in northeast China, as well as in parts of Japan, Korea, and Russia [2]. Differences in the nutrient content of *P. koraiensis* seeds are often caused by different water qualities, soils, climates, and latitude and longitude conditions [1]. The lipid in *P. koraiensis* seeds directly determines the special flavor and taste of the nuts, which are different from other nuts and one of the important agronomic benefits of the tree [1,2,3]. The lipid content is significant in the production of pine nut oil. The lipid components of *P. koraiensis* seeds include unsaturated fatty acids such as oleic acid and linoleic acid [3]. The unsaturated fatty acids in the seeds possess an anti-atherosclerotic effect and also enhance brain cell metabolism while maintaining brain cell function and nerve function [2]. At present, the traditional detection techniques of this species still rely on experienced staff to distinguish the origin of the plant [1,4]. The lipid content of pine nuts is difficult to detect by the naked eye; hence, manufacturers perform extraction tests on raw materials. Although the results obtained by extractions are more accurate, the process is destructive and time-consuming, and the special chemical reagents used (such as ether) are dangerous. If the waste liquid left after the experiment is not handled properly, it will pollute the environment [4]. Therefore, a non-destructive testing method is needed to accurately and quickly analyze the origin and lipid content of *P. koraiensis* seeds.

In the near-infrared (NIR) spectrum, the absorption of light by organic substances depends on the molecular overtone and combination vibrations of its hydrogen-containing groups (such as C-H, O-H, and N-H) [5]. Most of the overtone and combination vibrations of interatomic vibrations in molecules are in the NIR spectrum, so the NIR spectrum contains abundant information [6]. In addition to analyzing components associated with hydrogen-containing groups such as proteins, fats, and starch, it can also be used to analyze the density and mechanical properties of samples and elucidate other complex, unknown properties (unstudied properties) [6,7,8]. NIR spectroscopy (NIRS) has been used in the fields of food, materials, chemical engineering, medicine, and physiological diagnoses [9,10,11,12]. NIRS has become very popular in the past decade. For example, rapid non-destructive testing in fruit production lines with NIR spectroscopy has represented the sweetness, freshness, and quality of apples [13]. NIRS is applied to many characteristics of agricultural products, such as the quantification of protein content in sweet potato [14] and the prediction of the level of astringency in persimmon [15]. When it comes to the use of NIR in nuts, in addition to conventional properties (such as moisture content), NIR has also been used to discern textural characteristics of roasted pistachio kernels [16] and aspects of quality evaluation in Macadamia nuts [17]. NIR detection technology has been used for more complex work in nuts, including the characterization of the chemical morphology of areca nuts [18], the detection of mold-damaged chestnuts [19,20], and the discrimination of peanuts in bulk cereals [21].

The objectives of the study are to qualitatively assess the three varieties of *P. koraiensis* seeds from different places of origin (the Changbai, Yichun, and Heihe regions, respectively) and to quantify the lipid content of *P. koraiensis* seeds using regression analysis. At present, there is no research on using NIR spectroscopy to simultaneously and non-destructively analyze the shell and inner kernel of pine nuts, and the algorithms used for data processing and modeling are inflexible. It is difficult to adjust the strategy and optimize the algorithm according to the complexity of the target task. Therefore, the focus of subsequent research will be to adjust the amount of information contained in the input data for different modeling objectives, in order to improve the detection efficiency while ensuring high accuracy.

The current study investigates preprocessing and feature selection techniques to process NIR spectral data. A mathematical model was established by combining the classification labels and lipid content. Principal component analysis (PCA) was selected to reduce the dimensionality of the input data used to build the classification model. Wavelet compression techniques were used to process the data and compare the quality of the compression model and produce the best wavelet coefficient matrix. The wavelet coefficient matrix was optimized by the Monte Carlo and uninformative variable elimination (MCUVE) algorithm. The PCA–partial least-squares (PLS) and wavelet transformation (WT)–MCUVE–PLS calibration models were developed to predict the origin and lipid content of *P. koraiensis* seeds and were compared with other conventional methods.

## 2. Materials and Methods

### 2.1. Material Collection and Preparation

A 2 kg sample of pine nuts was randomly selected from Changbai Mountain in Jilin Province, and the Yichun and Heihe regions in Heilongjiang Province. Forty *P. koraiensis* seeds were randomly selected from the samples from the three places of origin, giving 120 in total. A removable label was pasted on the outside of each sample, indicating the origin and serial numbers (C1–C40, Y41–Y80, and H81–H120 from the Changbai, Yichun, and Heihe regions, respectively). The rest of the *P. koraiensis* seeds were shelled and the pine nuts were ground to powder using a grinder. The 40 samples (each 5 g) of *P. koraiensis* seeds from each place of origin were put into transparent, sealed bags. One side of the sealed bag was marked with the serial number. The sampling process was random. Before the NIR reading, the samples (the pine nut powder) were individually sealed, kept away from light, and stored for 24 h at 21 ℃ to reach a stable state.

### 2.2. NIR Spectrometer and Spectral Acquisition

**Figure 1:**
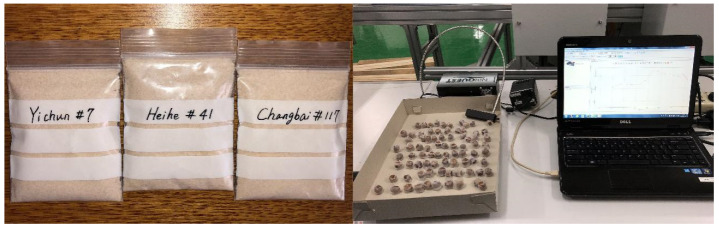
Pine nut samples and surface scan using the NIRQuest512 spectrometer.

The experimental data consisted of the NIR spectroscopy data and the lipid content analysis of the pine nuts. The NIR data were collected by a NIRQuest512 spectrometer (Ocean Optics, Inc., Dunedin, FL, USA), which was equipped with a fiber interface (SMA905) and detector (Hamamatsu G9204-512InGaAs linear array). The spectral grating selection of the instrument ranged from 900 to 1700 nm. During the NIR data reading, the measurement environment was maintained at a constant relative humidity and room temperature (fluctuating by no more than 1 ℃), and the distance between the probe and the sample was kept at 0.5 mm during measurement (Figure 1). The spectral data were determined by the mean of three measurements and collated using the configuration software program SpectraSuite (Ocean Optics, Inc., Dunedin, FL, USA). All the ground samples were properly preserved until chemical analysis.

### 2.3. Lipid Content

The lipid content of *P. koraiensis* seeds was determined using the Soxhlet extractor method as described by the AOAC [22]. The solvent used in the Soxhlet was anhydrous ether. The weighed filter paper bag was filled with 5 g of finely ground pine nut sample. The parts of the extractor were connected, the condensate water flow was connected, and the extraction was carried out at a constant temperature (70 °C). After extraction, the filter paper bag was removed and placed in a well-ventilated place while the ether volatilized completely before the sample was dried and weighed.

The crude lipid was calculated as follows:

(1) X=(b−c)/(b−a)×100%

where X is the crude lipid content; a is the weight of the weighing bottle and filter paper bag; b is the weight of the weighing bottle with the filter paper bag and drying sample; and c is the weight of the weighing bottle with the filter paper bag and the dry residue after extraction. The lipid analysis was carried out and certified by the Academy of Quality Supervision and Inspection in Heilongjiang Province.

### 2.4. Data Analysis

Standard normalized variable (SNV) aims to eliminate the effects of surface scattering, solid particle size, and light path changes during measurement on NIR diffuse reflectance spectra, which are generally the spectra used during data preprocessing in NIR spectroscopy. Uninformative variable elimination (UVE) and the successive projections algorithm (SPA) are widely used in the selection of spectral characteristic bands [15,23]. To address classification issues, the purpose of this study was to ensure the accuracy of classification while reducing the number of input features as much as possible; regression problems need to focus on optimizing data quality and preserving the integrity of data information [23]. Unlike traditional NIR stoichiometric analysis, in addition to SNV preprocessing, SPA, and UVE feature selection [24,25], this study specifically introduces wavelet transformation and wavelet compression techniques, the PCA method, and the MCUVE algorithm. On the basis of SNV, WT was used for further preprocessing of the data, which can optimize and compress the spectral data to obtain useful information. PCA can greatly reduce data size, and the MCUVE algorithm can improve the efficiency of feature selection. The PCA–PLS classification model and a better performing WT–MCUVE–PLS regression model were obtained as a result.

In this study, all the sample data were divided into calibration and prediction sets. Before each modeling scenario, the sample selection of each data set conformed to the principle of random sampling, but the numbers of the calibration and prediction sets were always fixed, corresponding to 80 and 40. In order to optimize the parameters and improve the prediction accuracy and generalization ability of the model, five-fold cross-validation was used to split the data set. In the process of modeling, the data set was divided into five parts randomly and evenly, four of which were used for training in turn, with the remaining part used for validation. For all the calibration models involved in this study, a grid-search was used to optimize the parameters. Within the specified parameter range, the parameters were adjusted in sequence according to the step length. The adjusted parameters were used to train the learner. All the mathematical models involved in this study were performed on MATLAB R2018a (The MathWorks, Natick, MA, USA).

#### 2.4.1. Discrete Wavelet Transformation

WT, which uses a wavelet basis function as a window function, transfers the time-domain signal to the wavelet domain coefficient through a wavelet basis function [26]. A discrete wavelet transformation (DWT) is any wavelet transformation for which the wavelets are discretely sampled, with the intention of selecting a discrete subset to be able to reconstruct a signal from the corresponding wavelet coefficients [27].

From an engineering point of view, the wavelet transformation is transformed into a set of filtering operations by the superimposition of the wavelet on the different initial time-domains [27]. The discrete wavelet transformation can be intuitively defined by Equations (2)–(4):

(2) $$c_{jk}=\nu \left(t\right),\varphi_{jk}\left(t\right)$$

(3) $$d_{jk}=\nu \left(t\right),\psi_{jk}\left(t\right)$$

(4) $$\nu \left(t\right)=\overset{\infty }{\underset{k=-\infty }{\sum }}c_{Jk}\varphi_{Jk}\left(t\right)+\overset{\infty }{\underset{j=J}{\sum }}\overset{\infty }{\underset{k=-\infty }{\sum }}d_{jk}\psi_{jk}\left(t\right)$$

where $c_{jk}$ and $d_{jk}$ represent the approximate wavelet coefficient and the detailed wavelet coefficient, respectively, and where J indicates the number of transformation levels, $\varphi_{jk}\left(t\right)$ is called the scaling function, and $\psi_{jk}\left(t\right)$ is called the wavelet function.

#### 2.4.2. Wavelet Threshold Denoising and Compression Method

In this study, a wavelet compression denoising algorithm was used for the extraction of the original spectral data features, reducing the original spectral data noise, and improving the accuracy of the model. Feature selection is crucial to the quality of the PLS calibration model [27]. Thus, wavelet compression can be added before modeling the wavelet coefficients to select specific wavelet coefficients for modeling. Although researchers in the NIR field use wavelet transformation as a common means to compress data sets, extract data features, and reduce noise [26,27], most do not pay attention to the wavelet transformation compression ratio, information loss, and final model quality. Two indices of compression ratio and information loss were used to evaluate the wavelet transformation.

At present, the mainstream wavelet denoising method is the wavelet threshold denoising method proposed by Donoho and Johnstone in 1994 [28]. Formally, Donoho and Johnstone defined the original signal and the noise signal in the wavelet denoising problem by Equation (5) [28]. If the one-dimensional signal is f(t), the signal can be expressed as (Equation (5)):

(5) $$f\left(t\right)=f_{s}\left(t\right)+f_{n}\left(t\right)$$

where $f_{s}\left(t\right)$ is the noise-free signal and $f_{n}\left(t\right)$ is the noise signal. The noise data have an i. i. d. Gaussian (Normal) distribution according to the Donoho and Johnstone studies, with a mean of zero and a variance $\sigma^{2}$, featuring a high frequency and small values. The wavelet transformation coefficients corresponding to the noise signal are almost all in the wavelet detail coefficients, and the coefficient values are low. Therefore, almost all the noise signals can be filtered by a suitable threshold algorithm.

#### 2.4.3. Principal Component Analysis (PCA)

Using the linear projection method [29,30] to reduce the dimensionality of the data [31], the aim of the PCA model is to make sure that the data projected in the given direction will produce the maximum variance (Equation (6)).

(6) $$\underset{\|w\|=1}{\max}Var\left\{w^{T}X\right\}$$

where X represents the data matrix. It is also equivalent to the minimum distance between the data point and its projection point (Equation (7)).

(7) $$\underset{W^{T}W=I}{\min}\overset{N}{\underset{n=1}{\sum }}\|x_{n}-WW^{T}x_{n}\|{}^{2}$$

where $x_{n}$ denotes the data points.

#### 2.4.4. Monte Carlo (MC) Combined with Uninformative Variable Elimination (UVE)

UVE algorithms are used for feature selection before modeling [25]. MC combined with UVE is designed to randomly split the original training data set into sub-data sets using MC algorithms and to implement the UVE–PLS model in the sub-dataset [25,26]. If the correlation coefficient matrix of spectral data is $\beta =\left[\beta_{1},\beta_{2},\cdots ,\beta_{p}\right]$, then stability can be judged by Equation (8):

(8) $$s_{j}=mean\left(\beta_{j}\right)/std\left(\beta_{j}\right)j=1,2,3,\cdots ,p$$

where $s_{j}$ is arranged in order from large to small, and the top k correlation coefficients are intercepted according to the threshold. The band corresponding to the top k correlation coefficients is the characteristic band selected by the MCUVE algorithm.

#### 2.4.5. Partial Least-Square (PLS)

PLS is often used to solve regression problems (partial least-squares regression; PLSR). A classification model can be established by adding a category determination step based on PLSR, which is called partial least-squares discriminant analysis (PLS-DA). The PLS algorithm is widely used in NIR spectroscopy for its stability [32,33,34,35]. In the lipid content regression model, for example, the lipid content matrix $Y={\left(y_{ij}\right)}_{n\times m}$ and absorbance matrix $X={\left(x_{ij}\right)}_{n\times xp}$ are decomposed into the form of feature vectors (Equations (9) and (10)).

(9) Y=UQ+F

(10) Y=TP+E

where U and T are the lipid content characteristic factor matrix and the absorbance characteristic factor matrix of n rows and d columns (d is the abstract group fraction), respectively. In addition, Q (d×m) is the lipid content load array, P (d×p) is the absorbance load array, and F (n×m) and E (n×p) are the lipid content residual array and absorbance residual array, respectively. Q is the load matrix of lipid content, P is the load matrix of absorbance, and F and E are residual matrices of lipid content and absorbance, respectively. The cross-validation method was used to obtain the value of d.

The PLS method decomposes Y and X according to the correlation of eigenvectors. The regression model was as follows (Equation (11)):

(11) $$U=\mathrm{TB}+E_{d}$$

where $E_{d}$ is a random error matrix and B is a d dimensional diagonal regression coefficient matrix. 

If the absorbance vector of the test sample is x, the lipid content is expressed as follows (Equation (12)):

(12) $$y=x{\left(UX\right)}^{\prime }\mathrm{BQ}$$

Unlike PLSR, PLS-DA uses a binary matrix as a response matrix instead of a numerical matrix [33]. There were three category vectors in this study, which were set to [0,0,1], [0,1,0], and [1,0,0].

### 2.5. Origin and Lipid Content Calibration Models

**Figure 2:**
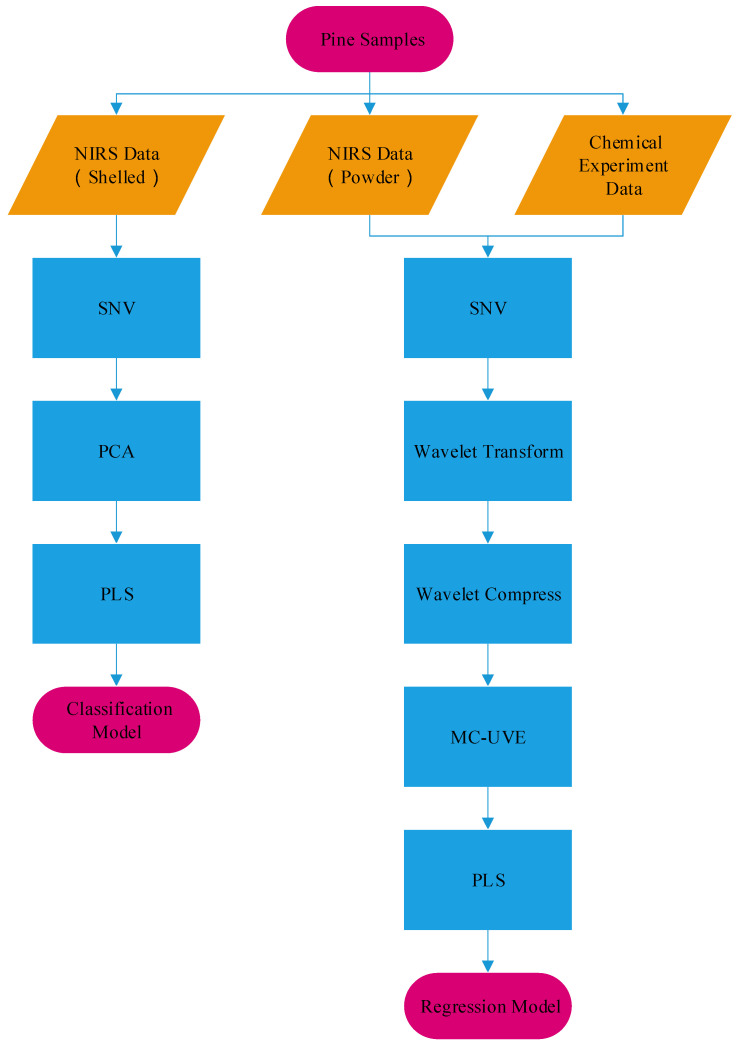
Flowchart of principal component analysis (PCA)–partial least-squares (PLS) classification and wavelet transformation (WT)–Monte Carlo and uninformative variable elimination (MCUVE)–PLS regression models.

The nonrelevant information in spectral data can compromise the precision and accuracy of the results [23,25,26]. The existing principle of NIR technology reveals that most regression calibration model inputs require only about 10 bands [23,25]. After wavelet compression, there is still space for compression and dimension reduction. Feature selection, used to extract useful information and eliminate irrelevant variables, is a crucial step before building the calibration model. In this study, PCA and WT–MCUVE were used for dimension reduction and feature transformation to produce the PCA–PLS classification model and the WT–MCUVE–PLS regression model (Figure 2).

### 2.6. Model Validation

The stability and reliability of the prediction model can be obtained by the correction parameters, such as the root mean standard error of cross-validation (RMSECV) and the root mean square error of prediction (RMSEP) [35,36]. More formally, the RMSECV and RMSEP for the NIR calibration model were computed by Equation (13):

(13) $$RMSECV/RMSEP=\sqrt{\frac{\sum_{i=1}^{n}{\left(y_{i}-{\hat{y}}_{i}\right)}^{2}}{n}}$$

where $y_{i}$ represents the measured value and ${\hat{y}}_{i}$ represents the predicted value. Furthermore, the correlation between chemical and spectral data is denoted by the $R^{2}$ of the calibration and prediction sets [37]. $R^{2}$ is computed by Equation (14):

(14) $$R^{2}=\sqrt{\frac{\sum_{i=1}^{n}{\left({\hat{y}}_{i}-y_{i}\right)}^{2}}{\sum_{i=1}^{n}{\left({\hat{y}}_{i}-\bar{y}\right)}^{2}}}$$

where $\bar{y}$ denotes average measurement. Percent root mean square difference (PRD) is used as a criterion for judging the degree of distortion of data [35], and can be expressed by Equation (15):

(15) $$\mathrm{PRD}=\sqrt{\frac{\sum_{j=1}^{n}{\left[x_{ori}\left(j\right)-x_{rec}\left(j\right)\right]}^{2}}{\sum_{j=1}^{n}x_{ori}{\left(j\right)}^{2}}}\times 100$$

where $x_{ori}\left(j\right)$ corresponds to the raw data, and $x_{rec}\left(j\right)$ corresponds to the reconstructed data (signal).

## 3. Results

### 3.1. Quantitative Analysis of Lipid Content

The chemical analysis of the lipid content of pine nut samples collected from the Yichun, Heihe, and Changbai Mountains are reported in Table 1. The lipid content of the sample is approximately 60%, and the difference in content depends on the temperature, humidity, soil conditions, and altitude of different growth environments [1,4]. In addition to satisfying the different environmental conditions, the three places of origin selected in this study also had different low-temperature periods in each year.

The descriptive statistics were expressed as standard deviation (SD), coefficient of variance (CV), mean, minimum (Min.), and maximum (Max.) values, as measured by the Soxhlet extractor method. The samples from Yichun had the highest lipid content, with an average value of 63.01%; the samples from Changbai Mountain had the lowest lipid content, with an average value of 60.90%. The highest and lowest values in the 120 groups of samples also came from the Yichun and Changbai Mountain, respectively. The mean, SD, and CV of the calibration and prediction sets of the final model were 61.90, 0.94, and 1.52, and 60.75, 0.71, and 1.16, respectively. The absolute value of the difference between the mean and extreme of samples ranged from 1.7% to 2%.

### 3.2. Spectral Data and Preprocessing Results

**Figure 3:**
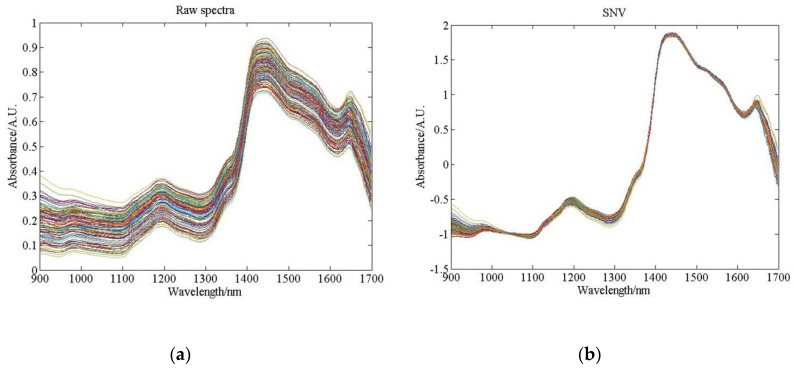
Near-infrared (NIR) raw spectra and standard normalized variable (SNV) pretreated spectra of whole pine nuts, (**a**) The raw spectra, (**b**) The SNV pretreated spectra.

Figure 3 shows the raw and SNV pretreated spectra of pine nuts with shells. The shell of pine nuts has a uniform texture and produces smooth spectra. During the measurement, it was difficult to ensure that the distance from the fiber probe to the pine sample was constant. The SNV method can effectively remove the regular differences caused by optical path changes.

**Figure 4:**
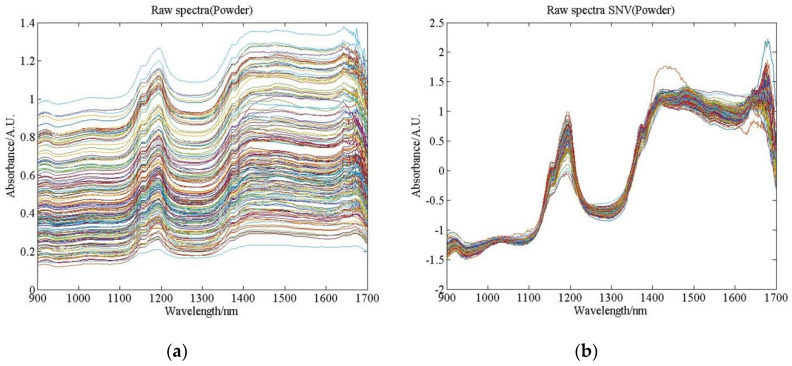

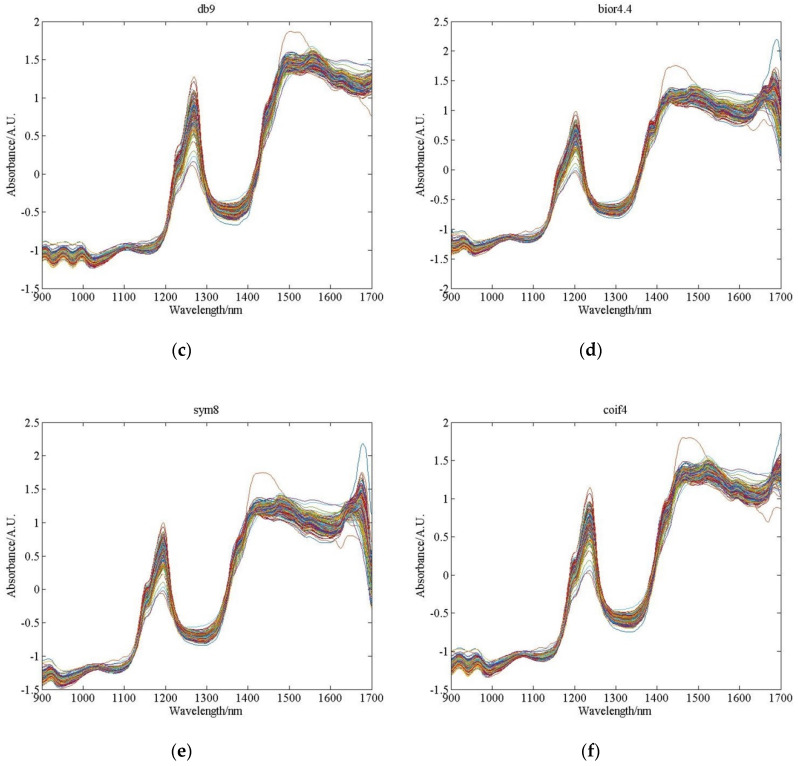
NIR raw spectra, SNV pretreated spectra, and wavelet compression spectra of pine nut powder, (**a**) The raw spectra, (**b**) The SNV pretreated spectra, (**c**) The spectra after being compressed by ‘db9’, (**d**) The spectra after being compressed by ‘bior4.4’, (**e**) The spectra after being compressed by ‘sym8’, (**f**) The spectra after being compressed by ‘coif4’.

Raw spectral data were collected from ground samples for regression analysis. After SNV processing, the data were compressed by the wavelet noise reduction threshold. Figure 4 shows the inverse transformation results after compression of the raw spectra, the SNV pretreated spectra, and the ‘db9’, ‘bior4.4’, ‘sym8’, and ‘coif4’ noise reduction thresholds. High-frequency noise was filtered out by reconstructing NIR spectral bands in ‘db9’. Compared with the original NIR spectroscopy, the spectra of ‘bior4.4’ and ‘sym8’ in the wavelength ranges of 1100–1200 nm and 1600–1700 nm subintervals were distorted.

In this study, the four wavelet basis functions of ‘db9’, ‘bior4.4’, ‘sym8’, and ‘coif4’ were used for three layer decomposition. The compression performance of four different wavelet compression methods: the Birge–Massart Strategy, SURE Shrink Thresholding, Donoho Thresholding, and Soft Thresholding, were tested. A performance optimal wavelet compression method was selected, and the PRD was used as the criterion to judge the degree of data distortion [38].

Table 2 shows the compression rate and the degree of data distortion of the four different wavelet compression methods. The performance of Soft Thresholding was too rough: data integrity from this method was the worst of all and the PRD ranged from 0.37% to 0.39%. SURE Shrink Thresholding and the Birge–Massart Strategy methods focus on improving the compression rate. The Donoho Thresholding method emphasizes data integrity, but the compression rate was the worst of all methods. In this study, the feature selection of wavelet coefficients was performed using MCUVE so that the desired effect of wavelet compression resulted in less data loss rather than a larger compression rate. This approach filtered out a small amount of high-frequency and low-amplitude information to reduce the noise effectively and make the spectral data smoother. Combined with the results of Table 2 and Figure 4, the compromise between data loss and the compression rate was regulated. According to the actual requirement of this study, the Donoho Thresholding with the ‘bior4.4’ wavelet filter was chosen to compress the NIR raw data, with a corresponding compression rate of 84.7487% and a PRD of 0.21%.

### 3.3. Results of Feature Selection

#### 3.3.1. Results of Principal Component Analysis (PCA)

**Figure 5:**
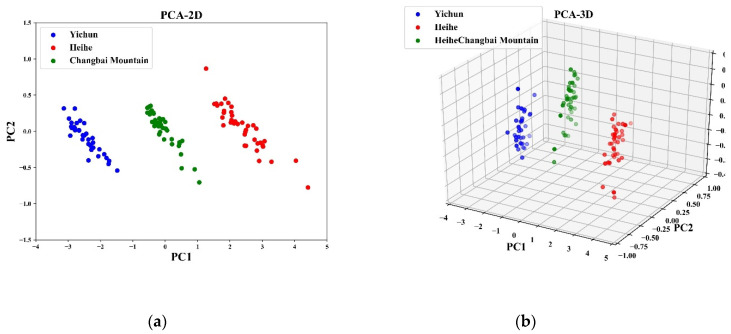
Score plots of the pine nut samples in the space defined by the first two and three principal components, (**a**) The visualization of PCA-2D, (**b**) The visualization of PCA-3D.

The data visualization output of reducing high-dimensional data to two and three dimensional data using PCA is displayed in Figure 5. When reduced to two dimensions by the PCA method, the three groups of samples were completely separated. When three principal components were produced, the three sets of sample data points were still not interleaved in three dimensional space. Comparing the two cases, the distance between data points of similar samples in three dimensional space was closer. By comparison of the data visualization effects, it can be concluded that the effects of reducing dimensionality by PCA to two and three dimensions were similar. Therefore, there is no need to choose more principal components.

#### 3.3.2. Results of Monte Carlo-Uninformative Variable Elimination (MCUVE)

**Figure 6:**
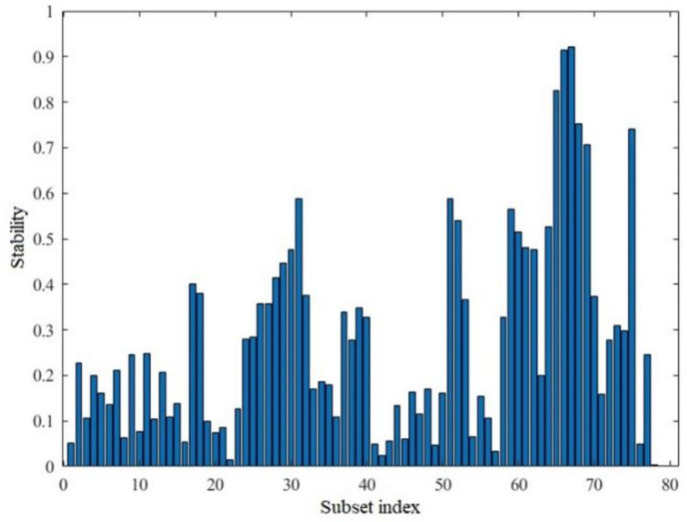
Stability diagram of spectral bands selected by MCUVE.

MCUVE was used for feature selection of wavelet coefficients. Figure 6 shows the results of the stability of subsets of spectral bands selected by MCUVE. The top 70 sets of wavelet coefficient data with the best stability were selected and used to establish a PLS calibration model of the relationship between the pine nut fruit lipid content and the NIR spectrum.

### 3.4. Model Results and Analysis

#### 3.4.1. Results of The Classification Model

Table 3 shows the output of each parameter in the calibration and prediction sets under the different classification schemes. The calibration model was evaluated with Precision, Recall, and F1 [30]. Values of the three indices are in the range of 0 to 1: the closer to 1, the better the performance of the model. The three indices of the SNV–PCA–PLS calibration model were better than the calibration model without PCA dimension reduction, which were 1.0, 0.94, and 0.97, and 0.97, 0.95, and 0.97, for Precision, Recall, and F1, respectively.

Combined with the results of Figure 5 and Table 3, when the input data were reduced by PCA to two and three dimensions, the accuracy of the calibration and prediction sets were the same and were the highest, at 98.75% and 97.5%, respectively. Thanks to the significant reduction in the dimensionality of the input data, the average training time and average prediction time were reduced to 2.61 s and 0.91 s, respectively. Although the choice of three principal components had a better Recall value, the distance between data points of similar samples was closer. From the perspective of improving modeling efficiency, it is evident that using the SNV–PCA–PLS scheme with two dimensional input data produced the best comprehensive performance of the classification model.

#### 3.4.2. Results of the Regression Model

**Figure 7:**
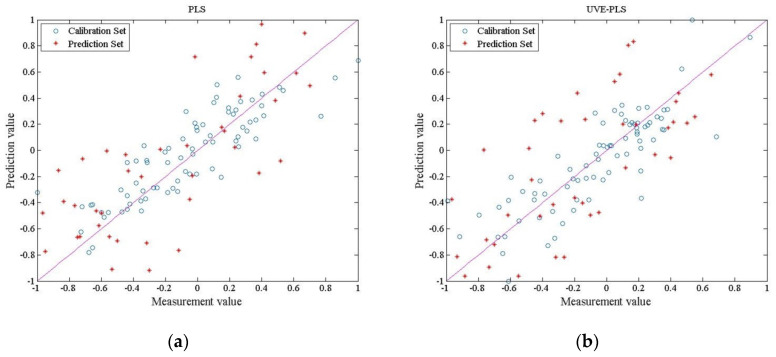

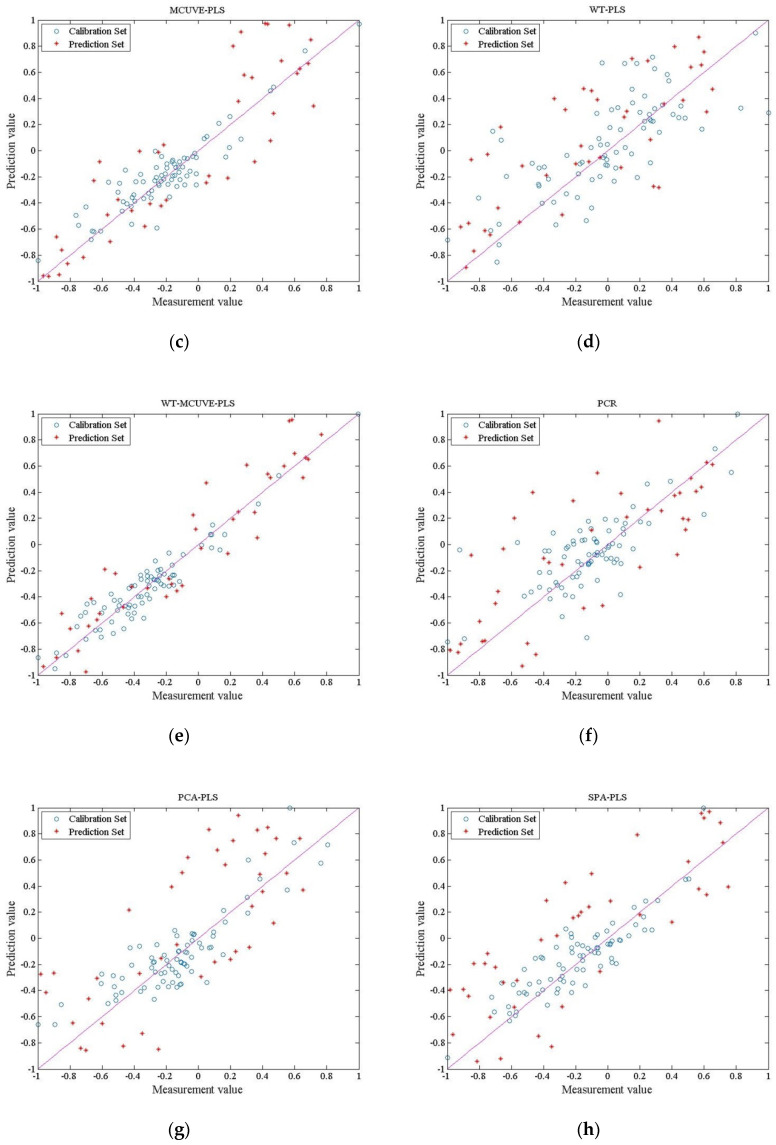
Correlation plot for the prediction of lipid content using PLS, UVE–PLS, MCUVE–PLS, WT–PLS, WT–MCUVE–PLS, principal component regression (PCR), PCA–PLS, and successive projections algorithm (SPA)–PLS models based on NIR spectra, (**a**) The visualization output of PLS, (**b**) The visualization output of UVE–PLS, (**c**) The visualization output of MCUVE–PLS, (**d**) The visualization output of WT–PLS, (**e**) The visualization output of WT–MCUVE–PLS, (**f**) The visualization output of PCR, (**g**) The visualization output of PCA–PLS, (**h**) The visualization output of SPA–PLS.

The RMSECV and RMSEP of each method and the correlation coefficient $R^{2}$ are shown in Table 4. The relationship between the observed lipid content and the results predicted by each calibration model is shown in Figure 7.

The differences in calibration model accuracy among the PLS, UVE–PLS, MCUVE–PLS, WT–PLS, WT–MCUVE–PLS, principal component regression (PCR), PCA–PLS, and SPA–PLS models are shown in Table 4 and Figure 7. The performance of the PLS model was worse than that of the MCUVE–PLS model. The implementation of the WT–MCUVE–PLS was better than that of the MCUVE–PLS model. The RMSECV and RMSEP of the PLS, MCUVE–PLS, and WT–MCUVE–PLS models were 0.0407, 0.1396, and 0.0449, 0.1556, and 0.0098, 0.0390, respectively. The WT–MCUVE–PLS model had the highest $R^{2}$ in the calibration and prediction sets of 0.9485 and 0.9369, respectively. It can be seen that the model has better accuracy and correlation when the input data undergoes the synergy of preprocessing and feature selection. By comparing the output of $R^{2}$, it can be seen that the performance of the WT–PLS model was worse than that of the UVE–PLS model. The RMSECV and RMSEP of the two models were 0.0808 and 0.1491, and 0.0159 and 0.0875, respectively. Since the input data of the UVE–PLS model was not pretreated and the number of features was reduced, the $R^{2}$ of its calibration and prediction sets was the second highest of all the models, and the distribution of data points in Figure 7 can be explained. The input matrix features of the eight models were 511, 100, 70, 154, 70, 511, 80, and 50 (Table 4). After a grid-search was used for global optimization, the MCUVE–PLS model had the best quality when the feature number of the MCUVE output matrix was 70. To achieve the best results, the feature number of the input matrix of WT–PLS was reduced to 154 by WT. Under the joint action of WT and MCUVE, the final feature number of the input matrix of WT–MCUVE–PLS was 70. The model performance gradually increased according to the decrease in input matrix features. Therefore, choosing a more efficient data compression scheme can significantly improve model performance. Of the above schemes, WT–MCUVE–PLS was the best model for predicting performance. In comparison with the other methods, the regression effect of PCR and SPA–PLS was good, but there was a certain gap compared with WT–MCUVE–PLS. PCA–PLS had a poor regression effect because of the limited amount of information carried after dimension reduction.

## 4. Discussion

In this study, the input data of the classification model preserved enough feature information for classification by PCA. The reduction in computation greatly reduced the time required for modeling and prediction, while improving the accuracy of prediction. In the process of establishing the regression model, wavelet transformation was applied to the processing of spectral data, and the MCUVE algorithm was used to select features. The WT–MCUVE–PLS model was superior to the traditional PLS, WT–PLS, and UVE–PLS in terms of computation, prediction accuracy, and time consumption. It had great advantages in comparison with the prediction effect of PCA–PLS, SPA–PLS, and PCR. The advantages were as follows: In the wavelet domain, DWT transformed discrete signals into approximate wavelet coefficients and detailed wavelet coefficients more quickly and conveniently. The data collected by the spectrometer are distributed in the range of 600–2400 nm in the form of discrete data points, which is the commonality of mainstream spectrometers [39,40,41]. Therefore, the discrete wavelet transformation is a good prospect in the field of NIR analysis, especially in the aspects of noise reduction and spectral data compression. Compared with using UVE to extract features from modeling data, MCUVE has several advantages. The MC algorithm aims to segment the original data set and the amount of subset data obtained is greatly reduced, thereby improving the efficiency of feature selection.

The algorithm in this study, combined with a portable NIR spectrometer, has practical significance for the production of *P. koraiensis* seeds and other kinds of nuts with shells. The characteristics of the food itself and the specific organic content can be quickly tested by analyzing the spectral data of the shells and kernels in combination with the actual chemical properties. The nutshell is the hard woody covering around the kernel of a nut. Both pine nutshells and wood contain a lot of cellulose, and the spectral curves are similar. The physical and chemical properties of pine nuts are similar to those of peanuts and other nuts. The qualitative analysis of wood by NIR technology and the quantitative analysis of nutrient content in foods, especially nuts, were used as important references for this study [7,36]. This technology is applicable in the classification of food and improves the related detection capabilities of spectroscopic equipment. The extrapolation of these analysis methods to the analysis of other foods is also highly relevant.

## 5. Conclusions

In this study, portable NIR spectroscopy was successfully combined with effective chemometric methods to classify and identify different places of origin of *P. koraiensis* seeds, and the lipid content of the inner kernel of *P. koraiensis* seeds was predicted by a regression model. Compared with traditional methods, the NIR detection method was more rapid, non-destructive, and environmentally friendly. Based on PCA, a PCA–PLS classification model of places of origin was established. The input data dimension was greatly reduced by PCA. Meanwhile, the prediction accuracy was improved and the time loss was reduced. From the results, it can be concluded that the best scheme was reduced to two dimensions by PCA. In the study of the lipid content prediction model, the compression effect of wavelet transformation on input data was discussed in detail. Combining MCUVE technology and PLS technology, a WT–MCUVE–PLS prediction model of lipid content was established. This method was efficient and accurate for the preprocessing and feature selection of input data. Compared with PCA–PLS, SPA–PLS, and PCR, the WT–MCUVE–PLS model had the best prediction results. Choosing wavelet compression technology reasonably and combining it with the MCUVE method can produce a more concise and effective model. Finally, this can achieve the onsite analysis of *P. koraiensis* seeds.

## Figures and Tables

**Table 1:** Descriptive statistics for lipid contents of *Pinus koraiensis* seeds as measured by Soxhlet extraction.

Set	Mean	Min ^(b)^	Max ^(c)^	SD ^(d)^	CV ^(e)^
Yichun#1–40	63.01	62.60	63.40	0.27	0.41
Heihe#41–80	61.17	60.30	62.20	0.52	0.85
Changbai Mountain#81–120	60.90	59.70	62.30	0.76	1.26
Calibration set (n ^(a)^ = 80)	61.90	59.70	63.40	0.94	1.52
Prediction set (n ^(a)^ = 40)	60.75	60.10	62.20	0.71	1.16
Total#120	61.70	59.70	63.40	1.09	1.77

^(a)^*n* sample number; ^(b)^ *Min* minimum; ^(c)^ *Max* maximum; ^(d)^ *SD* standard deviation; ^(e)^ *CV* coefficient of variation, CV = [{SD/Mean} × 100].

**Table 2:** Compression rate and data distortion of different wavelet compression methods.

Wavelet Filter	Threshold Methods	Compression R (%)	PRD ^(a)^ (%)
db9	Birge–Massart Strategy	85.1519	0.28
	SURE Shrink Thresholding	86.0656	0.36
	Donoho Thresholding	83.9738	0.23
	Soft Thresholding	86.0714	0.37
bior4.4	Birge–Massart Strategy	85.6925	0.28
	SURE Shrink Thresholding	86.7016	0.39
	Donoho Thresholding	84.7487	0.21
	Soft Thresholding	86.7525	0.39
sym8	Birge–Massart Strategy	84.6819	0.27
	SURE Shrink Thresholding	85.9911	0.36
	Donoho Thresholding	83.5233	0.20
	Soft Thresholding	86.1487	0.37
coif4	Birge–Massart Strategy	83.7562	0.26
	SURE Shrink Thresholding	85.2497	0.37
	Donoho Thresholding	82.5347	0.20
	Soft Thresholding	85.5128	0.38

^(a)^*PRD*, percent root mean square difference.

**Table 3:** Comparison of classification model results.

Model	Input Dimensions	Calibration Set					Prediction Set	
		Accuracy (%)	Time /s	Precision	Recall	F1 ^(a)^	Accuracy (%)	Time /s
PLS ^(b)^	511	78.75	8.96	0.85	0.93	0.81	77.50	4.46
SNV ^(c)^–PLS	511	88.75	8.13	0.93	0.95	0.92	87.50	3.32
SNV–PCA ^(d)^–PLS	2	98.75	2.61	1.00	0.94	0.97	97.50	0.91
	3	98.75	2.96	0.97	0.95	0.97	97.50	1.03

^(a)^ F1 = [{2 × Precision × Recall} / {Precision + Recall}]; ^(b)^ *PLS* partial least-squares; ^(c)^ *SNV* standard normalized variable; ^(d)^ *PCA* principal component analysis.

**Table 4:** Comparison of calibration results and prediction results obtained with the use of partial least-squares (PLS), uninformative variable elimination (UVE)–PLS, Monte Carlo (MC)–UVE–PLS, wavelet transformation (WT)–PLS, WT–MCUVE–PLS, principal component regression (PCR), principal component analysis (PCA)–PLS, and successive projections algorithm (SPA)–PLS models.

Model	Number of Features	Calibration Set (n ^(a)^ = 80)		Prediction Set (n ^(a)^ = 40)	
		RMSECV ^(b)^	R^2 (d)^ (Cal ^(e)^)	RMSEP ^(c)^	R^2 (d)^ (Pre ^(f)^)
PLS	511	0.0407	0.8613	0.1396	0.7489
UVE–PLS	100	0.0159	0.9169	0.0875	0.8810
MCUVE–PLS	70	0.0449	0.8369	0.1556	0.6721
WT–PLS	154	0.0808	0.7284	0.1491	0.7595
WT–MCUVE–PLS	70	0.0098	0.9485	0.0390	0.9369
PCR	511	0.0467	0.7512	0.1357	0.7540
PCA–PLS	80	0.0284	0.8635	0.1693	0.7330
SPA–PLS	50	1.6666	0.8820	0.1538	0.8141

^(a)^*n* sample number; ^(b)^*RMSECV* standard error of cross-validation; ^(c)^ *RMSEP* root mean square error of prediction; ^(d)^ *R^2^* multiple correlation coefficients; ^(e)^ *Cal* calibration set; ^(f)^ *Pre* prediction set.
